# Systematic Analysis of the *Pleurotus ostreatus* Laccase Gene (*PoLac*) Family and Functional Characterization of *PoLac2* Involved in the Degradation of Cotton-Straw Lignin

**DOI:** 10.3390/molecules23040880

**Published:** 2018-04-11

**Authors:** Xiaoyu Jiao, Guoqing Li, Yan Wang, Fan Nie, Xi Cheng, Muhammad Abdullah, Yi Lin, Yongping Cai

**Affiliations:** 1 School of Life Sciences, Anhui Agricultural University, No. 130, Changjiang West Road, Hefei 230036, China; jxy2015@ahau.edu.cn (X.J.); liguoqing1976@163.com (G.L.); wangyanahau@163.com (Y.W.); cxzp1114@163.com (X.C.); abdullahpadana@hotmail.com (M.A.); linyi1957@126.com (Y.L.); 2 Horticultural Institute, Anhui Academy of Agricultural Sciences, Hefei 230031, China; fan.n@163.com

**Keywords:** *Pleurotus ostreatus*, laccase gene, phylogenetic analysis, expression profiling, overexpression, lignin degradation

## Abstract

Fungal laccases play important roles in the degradation of lignocellulose. Although some *PoLac*s have been reported in several studies, still no comprehensive bioinformatics study of the *LAC* family in *Pleurotus ostreatus* has been reported. In this study, we identified 12 laccase genes in the whole genome sequence of *P. ostreatus* and their physical characteristics, gene distribution, phylogenic relationships, gene structure, conserved motifs, and cis-elements were also analyzed. The expression patterns of 12 *PoLac* genes at different developmental stages and under different culture substrates were also analyzed. The results revealed that *PoLac2* and *PoLac12* may be involved in the degradation of lignin and the formation of the fruiting body, respectively. Subsequently, we overexpressed *PoLac2* in *P. ostreatus* by the *Agrobacterium tumefaciens*-mediated transformation (ATMT) method. The transformants’ laccase activity increased in varying degrees, and the gene expression level of *PoLac2* in transformants was 2–8 times higher than that of the wild-type strain. Furthermore, the lignin degradation rate by transgenic fungus over 30 days was 2.36–6.3% higher than that of wild-type. Our data show that overexpression of *PoLac2* significantly enhanced the lignin degradation of cotton-straw. To our knowledge, this study is the first report to demonstrate the functions of *PoLac2* in *P. ostreatus*.

## 1. Introduction

Laccase is a kind of multi-copper polyphenol oxidase which belongs to the group of copper blue oxidase protein family (MCOs) and can catalyze the oxidation of various phenolic and non-phenolic compounds [1]. Other members of this family include ascorbate oxidases (EC 1.10.3.3), ceruloplasmin (EC 1.16.3.1), and ferroxidases [2]. The present study found that laccase is not only widespread in fungi and plants, but also some bacteria [3] and insects [4] can also secrete laccase. Fungal laccase plays an important role in the biological process of fruiting body development, pigment formation, and stress resistance. In addition, laccase, as an enzyme of broad substrate specificity in the lignin-degrading enzymes, plays an important role in the decolorization of textile dyes [5], pulp bleaching [6], and lignin degradation [7]. Fungal laccase generally contains four copper molecules, the mononuclear center T1 with one copper atom (type-1 Cu), and the trinuclear cluster (T2/T3) consisting of one copper atom (type-2 Cu) and two coupled copper atoms (type-3 Cu) [2]. Fungal laccases capture the single electron from the substrate, and the catalytic oxidation of the substrate is achieved by the coordinated transfer of electrons and the change of valence states between four copper ions. Compared with lignin peroxidase, its catalytic process does not require the participation of H_2_O_2_ [8], so it has a particular advantage in catalyzing the degradation of lignin.

*Pleurotus ostreatus* is a common edible white-rot fungus, which can be grown on a variety of agricultural lignocellulosic wastes [9]. Laccase genes are widely present in various basidiomycetes and are usually encoded by a gene family. The largest laccase family is from *Coprinopsis cinerea* that comprises up to 17 laccase genes [2]. In addition, the laccase gene families of many other basidiomycetes, such as *Flammulina velutipes* [10], *Ganoderma lucidum* [11], *Volvariella volvacea* [12], and *Pleurotus sajor-caju* [13] have been reported. The sequencing of the white-rot *P. ostreatus* genome is available at the JGI website (http://genome.jgi.doe.gov/PleosPC15_2/PleosPC15_2.home.html) [14,15,16]. Twelve putative laccase genes have noted in the *P. ostreatus* genome. As a matter of fact, because these databases are often based on sequence homology to infer the function of the gene, there may be some errors. Moreover, although some *PoLac* were reported in several studies, no comprehensive bioinformatics study of the *Lac* family in *P. ostreatus* has been reported. Up to date, only six *P. ostreatus* laccase isozymes have been biochemically characterized [17]. Based on the transcriptional analysis of *P. ostreatus* laccase genes, Pezzella et al. [18] suggest that among the produced *P. ostreatus* laccases, LAC10 (POXC) may play a major role in vegetative growth. The expression of fungal laccase is closely related to many factors, such as carbon source [13], nitrogen source, the stage of the life cycle of a fungus [10], and so on. In the existing reports, the regulation of laccase expression by ferulic acid and copper ions has been studied [13,18,19]. However, to the best of our knowledge, there is little information on the expression levels of *PoLac* genes of *P. ostreatus* strains cultivated in the cotton-straw solid medium. As of yet, the basidiomycetes laccase gene family’s role in the transcriptional regulation mechanism of lignin degradation is not so clear. The paralogous laccase copies within the same species may have specifically evolved to fulfill a variety of targeted functions [1], while gene function is often directly related to the structure. So, bioinformatic and expression analysis of the *P. ostreatus* laccase gene (*PoLac*) family were important in exposing the function and transcriptional regulation mechanism of the *PoLac* family.

At present, only a few of fungal laccase gene functions have been experimentally proven, and most of this research has focused on the degradation of different dyes. In 2013, Ryu et al. successfully overexpressed *Pblac1* in *P. brumalis* and found that transformation strains showed higher lignin degrading activity [7]. After that, Arimoto et al. [20] reported that the homologous overexpression of *Gtlcc3* provided *G*. *trabeum* with ligninolytic activity against Japanese cedar wood. Laccase expression is regulated by gene expression levels [21]. Thus, higher laccase genes expression level in *P. ostreatus* will probably increase the production of laccase and thus improve the utilization rate of straw materials. 

In this work, a laccase gene *PoLac2* which may be related to lignin degradation was cloned and successfully overexpressed in *P. ostreatus*. Our results showed that *PoLac2* is involved in the degradation of cotton-straw lignin. As far as we know, this is the first report to demonstrate the functions of *PoLac2* in *P. ostreatus*. The results of this study provide a new insight into how white-rot basidiomycetes accomplish lignin degradation and provides useful guidance for extending the application of *P. ostreatus* laccase.

## 2. Results

### 2.1. Genome-Wide Identification of Laccase Genes in P. ostreatus

A total of 166 putative laccase genes were found using BlastP against the *P. ostreatus* genome. But, there are many repetitive sequences. Thus, we deleted all repetitive sequences and removed the sequences without conserved amino acid domains of the laccase gene. Finally, 12 members of the laccase multigene family were identified in the whole genome of *P. ostreatus* (Table 1), which is same as the gene sequence that was previously annotated as laccase in the *P. ostreatus* genome [14]. The predicted characteristics of these 12 *PoLac* proteins are listed in Table 1. The twelve identified laccase genes contain 9–21 introns and encode proteins comprising 507–630 amino acids, the molecular weight of each protein was predicted, ranging from 55 kDa to 69 kDa. Theoretical isoelectric points (pI) of the 12 *PoLac* proteins ranged from 4.53 to 7.78. SignalP and TMHMM2.0 results, respectively, show that all of the twelve laccases have the signal peptide and no transmembrane region, which indicates that these laccases belong to the secretory protein. SOPMA results of the laccase proteins revealed that these proteins contained 11.44–22.38% α-helices, 27.14–34.77% extended strands, 9.02–12.57% β-turns, and 39.93–46.55% random coils (Table S1).

To determine the distributions of the twelve laccases in this study, the 12 *PoLac* genes were mapped onto *P. ostreatus* scaffolds based on the starting positions of the laccase genes within the scaffold (Figure S1). Scaffold 6 contained the largest number of *PoLac* genes, representing 58.33% of the total number. Moreover, scaffolds 2, 3, and 8 each contained only one *PoLac* gene. It was observed that most laccase genes were located at the tops or middle parts of the scaffolds. Especially, *PoLac1*, *4*, *6*, *9*, *10*, *11* gathered in high density at the top of scaffold 6. In contrast, *PoLac8* is located in the middle region of the scaffold.

### 2.2. Analyses of Phylogenetic Relationships, Gene Structures, and Conserved Motifs of PoLac Gene Family Members

**Figure 1:**
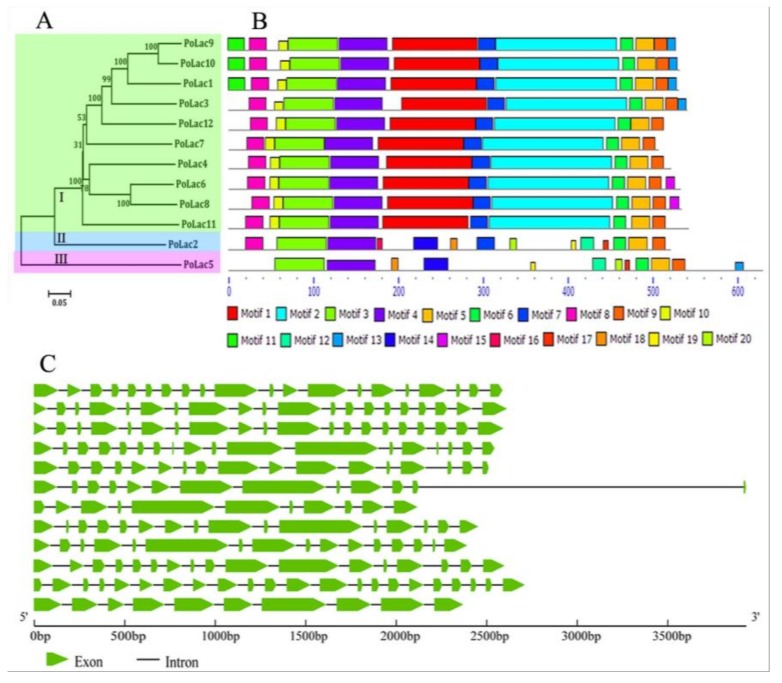
Phylogenetic analysis (**A**); conserved motif (**B**); and exon-intron structures (**C**) of *P. ostreatus* laccase gene family.

In order to analyze the phylogenetic relationship of *PoLac* genes, the phylogenetic tree was constructed based on PoLac1–PoLac12 with MEGA5.1 software using the neighbor-joining (N–J) method (Figure 1A). According to the results of the cluster analysis, the PoLacs were divided into three groups (I, II, and III). Group I contained the most PoLacs, PoLac2 and PoLac5 were separately clustered into group II and group III and exhibited low homology with others. The results of gene structures (Figure 1C) revealed that most *PoLac* genes clustered in the same group had similar exon-intron structures. For example, *PoLac1*, *PoLac9*, and *PoLac10* have the same number of introns. Furthermore, the nucleotide sequence of *PoLac2* is divided into more than 20 parts by introns, the largest number of introns in the 12 *PoLac* genes, indicating that the gene structure of *PoLac2* is more complicated than other *PoLac* genes. These results demonstrated the presence of highly conserved structures within the same groups and high sequence diversity among the different groups [22].

Twenty conserved motifs were identified in the *PoLac* proteins using the MEME web server (Figure 1B). The results show that most of the closely related *PoLac* proteins contained similar motif compositions, indicating that there are functional similarities between *PoLac* proteins within the same group. Subsequently, each conserved motif was analyzed using the Pfam database and SMART, with which the Cu-oxidase, Cu-oxidase 2, and Cu-oxidase 3 domains were respectively found in 6 motifs. Detailed information of the 20 conserved motifs is shown in Supplementary Table S2. Motif 15 was present only in *PoLac6* and *PoLac8*, which might be required for specific functions. Furthermore, some motifs, such as motifs 3, 4, 5, 6, and 9, were present in all *PoLac* proteins, reflecting their importance to the functions of these proteins.

### 2.3. Analyses of Multiple Sequence Alignment and Promoter Sequence

**Figure 2:**
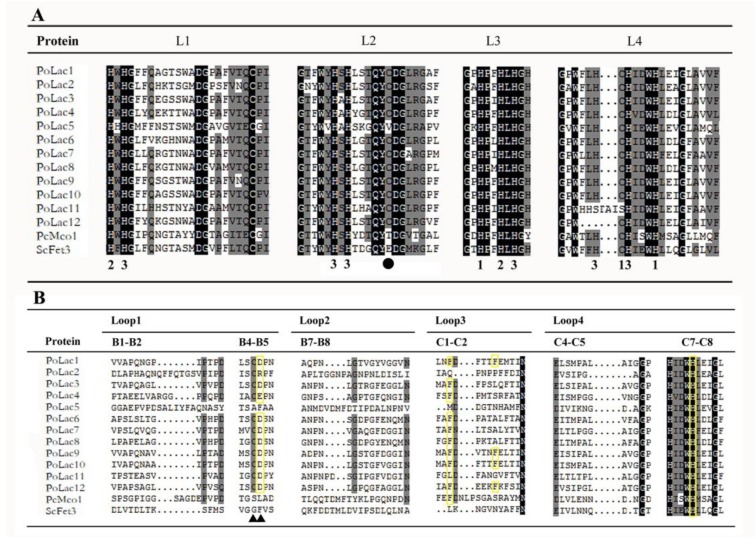
The signature sequences (**A**) and the postulated substrate binding loops (**B**) of *P. ostreatus* laccases Lac1 to Lac12 with the corresponding sequences from laccase Mco1 of *P. chrysosporium* and from fungal ferroxidase (Fet3) of *S. cerevisiae.* The cysteine (C) and the histidine (H) residues involved in copper binding are numbered according to the copper type (1, 2, and 3 for type 1, type 2, and type 3, respectively) they bind [23]. The black circle marks the cysteine (C) residue which is always present in classical laccases [23]. The sequences of potential substrate binding loops of the laccase enzymes identified according to Loops I–IV of laccase given in Figure 2 by Pezzella et al. [17] and according to the nomenclature of Hakulinen et al. [25]. Loops B1–B2 are located in the primary *PoLac1* sequence at positions 184–196, B4–B5 at positions 232–236, B7–B8 at positions 359–304, C1–C2 at positions 359–371, C4–C5 at positions 412–423, and C7–C8 at positions 482–491. Amino acids marked with a yellow box indicate those residues that correspond to amino acids shown in lac3b of *Trametes versicolor* to contact the substrate 2,5-xylidine [24]. Filled triangles underneath the sequences indicate residues in the B4–B5 β-hairpin loop that in the classical laccases are a highly conserved cysteine (C) and either an aspartic acid (D) or a glutamic acid (E) shown in lac3b of *Trametes versicolor* to contact organic substrates [25].

The amino acid sequence alignment of laccase genes from *P. ostreatus* and other basidiomycetes is shown in Figure 2A. As reported by Kumar et al. [23], the fungal laccase signature sequences L1–L4 have ten conserved histidines and one conserved cysteine of the copper-binding centers. Compared to the defined signature sequences by Kumar et al. [23], all predicted amino acid sequences contained complete laccase signature sequences L1–L4 except *PoLac5*, *PoLac11*, and *PoLac12*. A cysteine to serine change occurs in L4 of *PoLac11* and a histidine residue of *PoLac12* is absented in L4 of *PoLac12*. It is noteworthy that a cysteine to valine change occurs in L2 of *PoLac5*. The amino acid sequence lacking this conserved cysteine residue in signature sequence L2 is often similar to the ferroxidase amino acid sequence in the copper oxidase family [2].

All 12 proteins have substrate binding loops (Loops 1–4 in Figure 2B) described from three-dimensional structure analysis of crystallized laccases [24,25]. Structural analysis revealed protein–ligand interactions with specific residues in the pocket formed by the substrate binding loops [24]. It can be seen from Figure 2B that the number of substrate binding sites of PoLac1, PoLac9, PoLac10, and PoLac12 was higher than other PoLacs, while PoLac2 and PoLac5 had the fewest binding sites. These results suggest that there may be some differences in their ability to contact substrates. In the β-hairpin loop B4–B5, fungal laccases typically have a cysteine or an aspartic acid or glutamic acid [2]. A cysteine in B4–B5 is found in all *P. ostreatus* laccases but not in PoLac5 (Figure 2B). Combined with the above analysis of the signature sequence, PoLac5 may not be a typical laccase, and may be a ferroxidase in the family of multicopper oxidases (MCOs). The substrate binding loops of PoLac2 and PoLac5 were significantly different from other sequences. And this result is consistent with those of phylogenetic analysis.

**Figure 3:**
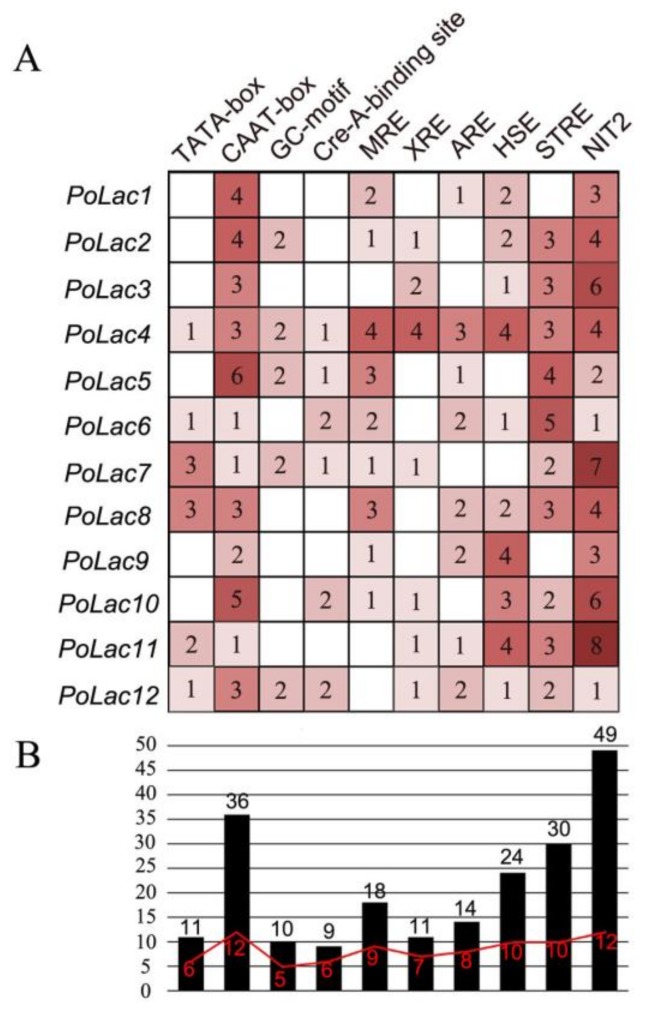
The number and the kind of cis-acting elements in the promoter regions of *PoLac*s. (**A**) putative cis-acting regulatory elements in the *PoLac*s promoters; (**B**) the number of cis-acting elements in the *PoLac*s promoter. And the column graph represents the total number of various cis-elements in the promoters of the *PoLac* genes, and the fold line indicates the number of genes containing the corresponding cis-elements in promoter regions.

To further explore the function and regulatory patterns of *PoLac* genes, a 2000-bp region of the genomic DNA sequence of each gene was analyzed and presented in Figure 3. The promoter regions of *PoLac1*–*PoLac12* include various cis-acting elements. In addition to the core promoter elements (TATA-box and CAAT-box) of eukaryotes, there are many other cis-acting elements, such as CreA-binding site [26], metal responsive element (MRE) [27], xenobiotic responsive element (XRE) [17], antioxidant responsive element (ARE) [17], heat shock sequence elements (HSE) [28], nitrogen binding site (NIT) [29], and a stress-responsive element (STRE) [30]. Moreover, the total number of NIT2 elements in the *PoLac* genes is the largest. On the contrary, the total number of CreA-binding site elements is the lowest. Although the different *PoLac* genes have different cis-acting elements in terms of number and variety, the presence of these cis-acting elements indicates that *PoLac* genes transcription can be regulated by metal ions, various aromatic compounds related to lignin or lignin derivatives, nitrogen, and carbon sources.

### 2.4. Laccase Activity of P. ostreatus and Degradation of Lignin from Cotton-Straw Medium by P. ostreatus

**Figure 4:**
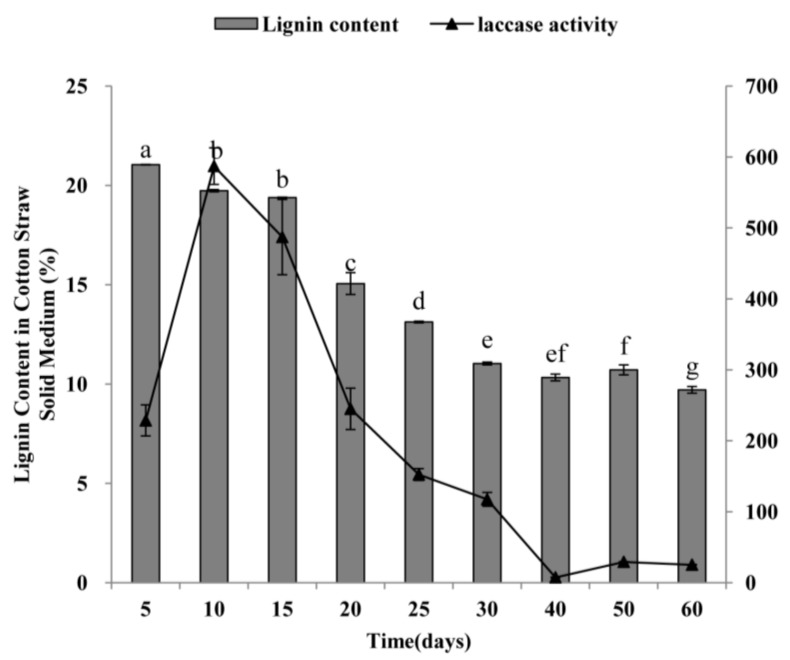
Evolution of the lignin content and laccase activity in solid-state fermentation (SSF) cultures of cotton-straw. The lowercase letters show a significant difference in lignin content (*p* < 0.05). Values are means of three replicates ± SD.

*P. ostreatus* was grown in the solid medium of cotton-straw for 60 days. The laccase activity and lignin content were measured to investigate the relationship between the laccase activity of *P. ostreatus* and degradation of lignin from cotton straw. The lignin content of the treated lignocellulosic substrates showed a decreasing trend during the 60 days of incubation (Figure 4). In the mycelium period (the first 30 days of cultivation), the lignin content was gradually reduced and was not obviously decreased in the first 15 days, whereas lignin content decreased significantly after the substrate was treated for 15 days. Especially on the 20th day, the content of lignin in the substrate decreased by 4.33% compared with the 15th day, accounting for 38.20% of the 60 days’ total degradation. In the fruiting body period (After incubation of the first 30 days), compared with the results of the 30th day, the lignin content of the treated substrates had changes only a little. As shown in Figure 4, the extracellular laccase activity was high in the early stage of the mycelial period and reached maximal levels within 10 days of incubation. After that, it declined sharply, especially after 15 days. Laccase activity remained low during the period of the fruiting body, and reached its lowest point at 40 days’ incubation. Thus, the results in Figure 4 suggest that there is a certain correlation between laccase activity and lignin degradation. The degradation of lignin from cotton straw was mainly during the mycelium stage. Therefore, laccase produced during the initial degradation stage might be primarily responsible for lignin depolymerization.

### 2.5. PoLac Gene Expression in Different Culture Substrates

**Figure 5:**
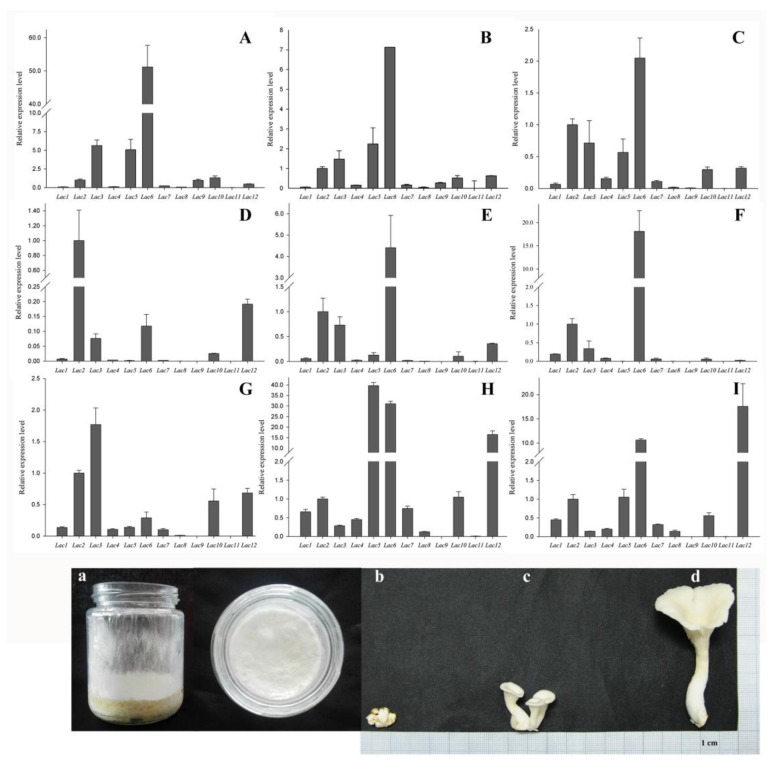
The relative expression of 12 *PoLac* genes at different developmental stages in the different culture medium. (**A**–**C**) the transcriptional profile of 12 laccase genes in the PDA medium during mycelium period (10 days, 20 days, and 30 days, respectively); (**D**–**F**) the transcriptional profile of 12 laccase genes in the solid medium of cotton-straw during mycelium period (10 days, 20 days, and 30 days, respectively); (**G**–**I**) the transcriptional profile of 12 laccase genes of primordium, juvenile, and maturation mushroom under cotton straw culture, respectively. Values are means of three replicates ± SD. And the figure correspondingly shows the samples from different growth stages of *P. ostreatus*. (**a**) the mycelium sample; (**b**) the primordium sample; (**c**,**d**) fruiting body under two growth stages (young and maturation), respectively.

The patterns of gene expression are often closely related to their functions. The transcription level of 12 laccase genes from *P. ostreatus* that grew on two different culture mediums were detected by real-time quantitative PCR (qRT-PCR) to further expose the laccase genes that may participate in the degradation of cotton-straw lignin. As shown in Figure 5, the *PoLac9* gene was not detected in any conditions of *P. ostreatus* growth on the solid medium of cotton-straw. However, the expression of *PoLac9* was detected in PDA medium conditions, with very low expression levels. The expression pattern of the *PoLac* family is varied. Under two different medium conditions, almost no expression of *PoLac4*, *PoLac7*, *PoLac8*, and *PoLac11* was observed. In cotton-straw medium cultures, the relative expression levels of *PoLac2* were significantly higher than those of any other laccase gene at the prophase of mycelium growth (10 days), and gradually decreased from the 20th day. The expression level of *PoLac6* increased significantly at 20th day and continued to rise until the 30th day. In the primordium and fruiting bodies, the expression of laccase gene is more abundant than in the mycelia period. *PoLac3* exhibited obviously preferential expression in the primordium. Therefore, this gene was inferred to be associated with the formation of the primordium. The relative expression of *PoLac5* was increased in the young fruiting body and then decreased in the mature fruiting body, indicating that this gene may be related to the formation of the fruiting body. *PoLac12* was significantly up-regulated during the formation of primordia and fruiting bodies.

### 2.6. The Lac2-Overexpressing P. ostreatus Transformants

**Figure 6:**
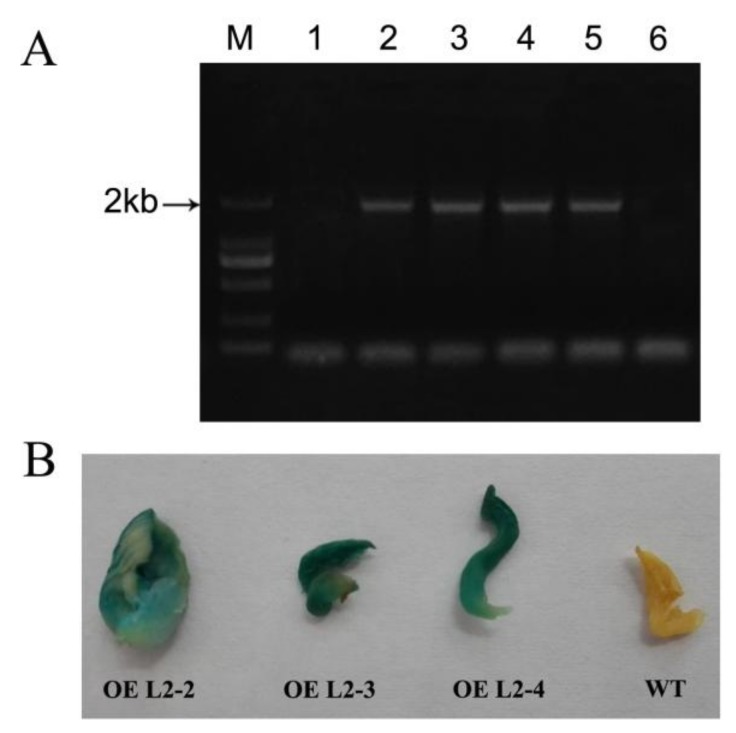
PCR identification and β-glucuronidase (GUS) staining of randomly selected *P. ostreatus* transformants and wild-types. (**A**) PCR identification of *gusA* gene, M: 2kb DNA marker, Lanes 1–5: Putative transformants (OE L2-1 to OE L2-5), 6: The non-transformed transformants as negative control; (**B**) GUS staining of the wild-type (WT) and three transformed strains (OE L2-2 to OE L2-4).

To investigate whether the foreign DNA fragments were integrated, five hygromycin resistant (Hyg^r^) transformants were randomly selected and analyzed for the presence of the β-Glucuronidase gene (*gusA*) using PCR (Figure 6A). The results of PCR revealed that 4 of the 5 transformants produced the expected PCR amplification, while *gusA* was not detected in the untransformed original strain. This indicated that *gusA* had successfully transferred into the genomic DNA of *P. ostreatus*. Subsequent GUS histochemical assays showed that the transformants have GUS activity, while no GUS staining was observed in the untransformed strain (Figure 6B). This result further indicated that the host chromosome received T-DNA.

### 2.7. Laccase Activity and Degradation of Lignin in Cotton-Straw Medium by Transformants

**Figure 7:**
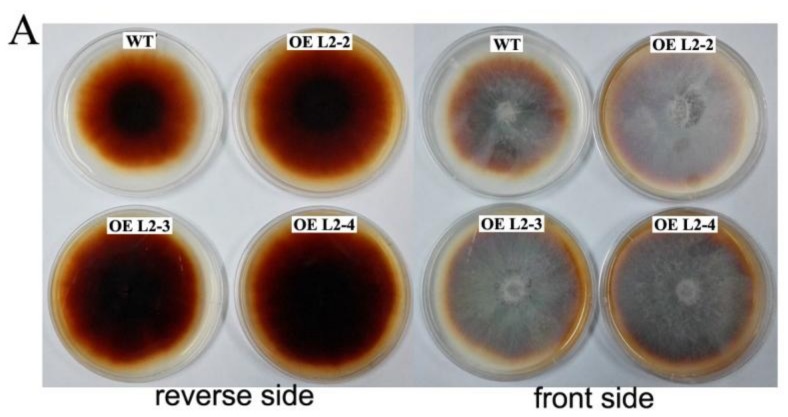

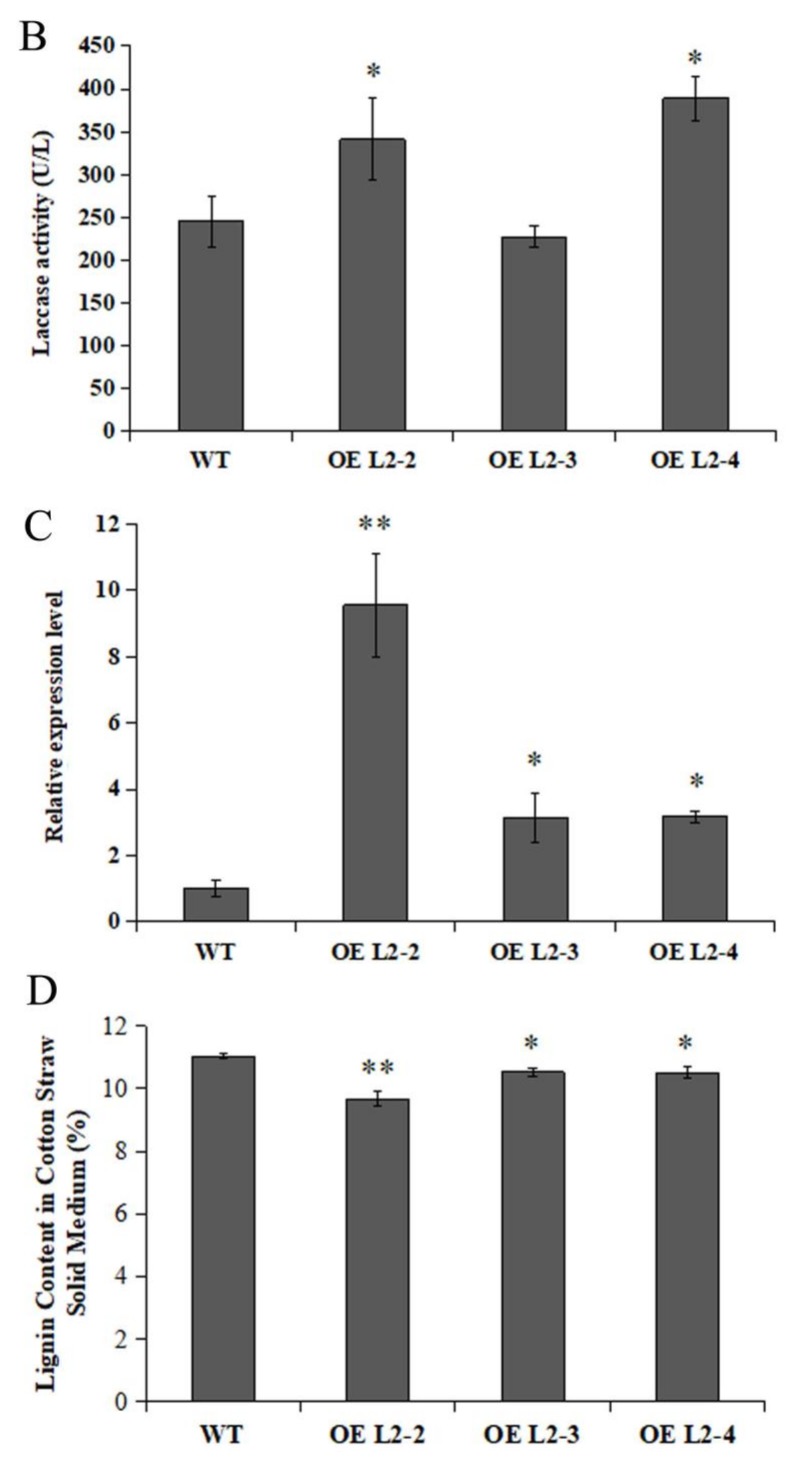
Comparative analysis of wild-type (WT) and randomly selected *P. ostreatus* transformants (OE L2-2, OE L2-3 and OE L2-4). (**A**) Laccase activities of the wild-type and transformants determined by guaiacol color-changing method; (**B**) Laccase activities produced of wild-type and transformants in cotton-straw medium; (**C**) Analysis of qRT-PCR for the *PoLac2* expression of the wild-type and transformants; (**D**) The lignin degradation rate of the wild-type and transformants cultivated in the cotton-straw medium after 30 days. * Denotes significant difference compared to wild-type (*p* < 0.05), ** Denotes extremely significant difference compared to wild-type (*p* < 0.01). The values are the means ± standard deviation (*n* = 3).

The transgenic strains were cultured on PDA plates supplemented with guaiacol for 7 days in an attempt to qualitatively analyze the laccase activity. As shown in Figure 7A, a clear crimson discoloration can be observed in guaiacol plates that cultured wild-type strains and transgenic strains. However, the transformants exhibited darker discoloration and longer colored-halo diameter than the wild-type strain. In addition, the extracellular laccase activity of two transformants (OE L2-2, OE L2-4) grown in the cotton-straw medium for 20 days was significantly higher than that of the wild-type strain (Figure 7B). Also, we detected the expression of *Lac2* in the three transformants by using qRT-PCR (Figure 7C). The results show that the *Lac2* expression in the three transformants was increased by approximately 2–8 times that of the wild-type strain, and especially transformant OE L2-2 had exhibited significantly increased expression. The degradation rate of cotton-straw lignin was detected and the result is shown in Figure 7D. The lignin degradation rates of the three transformants for 30 days were 54.92%, 50.92%, and 50.96% respectively, which was 6.3%, 2.36%, and 2.4% higher than that of wild-type, respectively, indicating that these transformants enhanced the degradation rate of cotton-straw lignin.

## 3. Discussion

With the development of genome-wide sequencing technology, the availability of fungal genome data is increasing [31]. Genome sequencing of *P. ostreatus* was completed in 2011. Although some *PoLac*s have been reported in several studies, no comprehensive bioinformatics study of the *Lac* family in *P. ostreatus* has been reported. In this study, 12 *PoLac* genes were identified and classified into three groups, which is in agreement with the gene sequence that was annotated as laccase in the *P. ostreatus* genome at the JGI portal. The 12 *PoLac*s were unevenly distributed among 5 scaffolds and clustered on scaffold 6 and scaffold 11 (Figure S1). Existing reports have shown that it is common for fungal laccase genes to be clustered on the scaffold. For example, *lac1*–*lac10* of the 11 laccase genes in the genome of *V. volvacea* strain V23 are centrally distributed on scaffold 6, while only *vv-lac11* is on scaffold 8 [32]. Moreover, the 17 laccase genes of *Coprinopsis cinerea* were distributed in 7 Contigs [2]. The main cause for this phenomenon is that in the long evolutionary process, the original laccase gene is differentiated into paralogous genes with different functions to fulfill the various functional requirements of fungi throughout the life cycle [23]. The result of two different fungi orthologous analysis showed that there are 11 orthologous genes in *Pleurotus ostreatus* and *Coprinopsis cinerea* (Table S4)*,* inferring that these genes may come from a common ancestor. The characteristic analysis results of the proteins in this report showed that all of the 12 laccases have the signal peptide and no transmembrane region, indicating that these laccases belong to the secretory protease. The fungal laccase amino acid sequence generally contains a signal peptide sequence at the N-terminus to guide transmembrane transfer. Nevertheless, it has been reported that *lac3* of *Flammulina velutipes* encodes an intracellular enzyme which does not contain a signal peptide sequence [10].

The result of gene structure analysis (Figure 1C) revealed that most *PoLac* genes clustered in the same group had similar exon-intron structures. A similar result occurred in the study of Kilaru et al. [2]. *PoLac2* and *PoLac5* were singly clustered into group II and group III, exhibiting low homology with the others. The genetic structures of *PoLac2* and *PoLac5* are also different from that of other *PoLac* genes. The number and distribution of introns are related to gene evolution [33]. In addition, the more similar the species, the more introns with the same position are observed, which may be due to the change of natural selection pressures after species divergence, leading to intron insertion or deletion [2].

Although the amino acid sequences of different laccase sequences are not identical, the signature sequences of the laccase genes exhibited high consistency. There are four conserved laccase signature sequences according to the literature [23]. Most notably, a cysteine to valine change occurs in L2 of *PoLac5*. Previous studies have shown that proteins lacking the conserved cysteine residue showed weak laccase activity but had strong ferroxidase activity [34]. This result implies that *PoLac5* is not a typical laccase and may be a ferroxidase of the copper oxidase family (MCOs). The 10th amino acid from the side of the cysteine residue (C) in signature sequence L2 determines the reduction potential of type I copper. A large hydrophobic group at this position has the stronger reduction potential [35]. According to the different types of amino acid residues in this position, the laccase has different reduction potentials. The highest reduction potential appeared when the amino acid residue is phenylalanine (Phe, F), followed by leucine (Leu, L), and methionine (Met, M) was the lowest [35]. As shown in Figure 2A, the amino acid residue of *PoLac1*–*PoLac5*, *PoLac9*, *PoLac10*, and *PoLac12* at this position is Leu, indicating that the catalytic ability of these eight laccases is moderate. The amino acid residue of *PoLac6*–*PoLac8* and *PoLac11* at this position is Phe, providing a strong reduction potential and indicating that their catalytic capability might be stronger than other *P. ostreatus* laccases. The first histidine residue in the copper binding site of T1/T3 in the signature sequence L4 of *PoLac12* is absent, which may affect the transfer of electrons in the oxidation reaction [36]. The analysis of the substrate binding loops’ sequences further demonstrated that PoLac5 may be a ferroxidase. Although PoLac2 did not have either an aspartic acid (D) or a glutamic acid (E) in the β-hairpin loop B4–B5, it did have a cysteine acid in the β-hairpin loop B4–B5 (Figure 2B) that interacts with organic substrates [24,25]. There are many cis-acting elements in the nucleotide sequences extending 2000 bp upstream of the start codons of *PoLac*. These include metal response element (MRE), stress responsive element (STRE), nitrogen binding site (NIT), etc. These cis-acting elements are widely found in fungal laccase genes and play a significant role in regulatory process. As shown in Figure 3, the promoter regions of all *PoLac* genes have the metal-responsive element except *PoLac3*, *PoLac11*, and *PoLac12*. The report on *P. ostreatus* pointed out that adding copper ions can enhance the transcription level of the laccase gene [37]. This conclusion is also reported in other species, such as *Trametes velutina* [38] and *Ceriporiopsis subvermispora* [39]. The addition of small aromatic molecules with similar structures to lignin in the culture medium could increase the yield of laccase. The main reason is that the aromatic compound can form a heterodimer with the nuclear-translocated protein, then bind to the xenobiotic response element (XRE), activating the transcription of the target gene. XREs have also been found in *Pleurotus sajor-caju* [13] and *Volvariella volvacea*. The potential CreA-binding site motifs were identified in the promoter of *PoLac2*–*PoLac4*, *PoLac7*, and *PoLac10*–*PoLac12*, suggesting that these laccase genes can be regulated by aromatic substances. Interestingly, The NIT2 element exists in all 12 *PoLac* promoters, and the total number of NIT2 elements in the *PoLac* genes is the largest. The result indicates that NIT2 elements may have a greater impact on the expression of these laccase genes.

The elucidation of gene expression patterns can provide important clues regarding gene functions [22]. In this study, we analyzed the relative expression of 12 laccase genes at different developmental stages of *P. ostreatus* that were grown in a cotton-straw medium as the sole carbon (and nitrogen) source. The result of the lignin content of the treated lignocellulosic substrates indicated that lignin degradation in lignocellulose mainly occurred at the mycelial growth stage, but not at the fruiting body stage. The trend of laccase activity suggested that laccase produced during the initial degradation stage might be primarily responsible for lignin depolymerization (Figure 4). *PoLac2* showed low expression levels in PDA culture, while cotton-straw culture significantly induced the expression of *PoLac2* at the prophase of mycelium growth. This suggests that *PoLac2* is closely related to lignin degradation. *PoLac6* has a high level of expression in PDA culture with high concentrations of glucose (Figure 5A–C), while it has a low level of expression at 10th days of mycelium grown in cotton-straw culture (Figure 5D). Then, the expression level of *PoLac6* increased significantly at 20th day and continued to rise until 30 days. The degradation of lignocellulose leads to a change in cotton-straw culture, the increased content of easily absorbed carbon affects the expression level of *PoLac6. PoLac12* was significantly up-regulated during the formation of primordia and fruiting bodies. This result is consistent with the study of Lettera et al. [40]. This is the first speculation that *PoLac3* and *PoLac5* may play a specific role in the formation of primordia and juvenile fruiting bodies. Most notably, the *PoLac9* gene was not detected in any of the tested conditions of *P. ostreatus* growth on cotton-straw. This does not agree with the result that *PoLac9* was strongly overproduced in lignocellulose cultures [41]. These results provide information that may facilitate further functional analyses of *PoLac* genes.

In fungi, laccase is involved in a variety of cellular physiological events [42]. The gene expression profile analysis can help us to infer the possible functions of some genes. Our above work regarding the transcription level suggested that the *PoLac2* gene may be closely related to the degradation of lignin. Published studies have indicated that the transcription of *PoLac2* and *PoLac10* were highly expressed in submerged fermentation cultures with wheat straw extract, at the same time, it is also the main source of laccase activity under submerged cultures [43]. Also, research on the secretome of *P. ostreatus* growth on the woody and nonwoody substrates have shown that LACC2 was strongly overproduced in the lignocellulose cultures [41]. In order to verify whether the *PoLac2* gene is associated with the degradation of lignin, we overexpressed *PoLac2* in *P. ostreatus* by ATMT mediated transformation. The results shown in Figure 6 indicated that the T-DNA fragments were integrated into the chromosomes of the transformants. It is known that the complex turns crimson when guaiacol is oxidized by the activity of laccases or peroxidases [44]. In previous reports, guaiacol was often used to detect the laccase activity of fungi [44]. In this study, the transformants exhibited darker discoloration and longer colored-halo diameter than the wild-type strain (Figure 7A). This result indicated that the transformants might have increased lignin-degradation activity. However, it is worth noting that this can only be used as a qualitative analysis. The color and diameter of the colored-halo do not completely represent the vitality of the laccase and enzyme activity needs to be determined to quantitatively analyze whether the enzyme activity really increased. As compared with the wild-type strain, the transformations have higher laccase activity, relative expression of *PoLac2* gene, and lignin degradation rate (Figure 7B–D), especially transgenic strains OE L2-2, which showed the highest ligninolytic activity among all the randomly selected transformants. These results revealed that *PoLac2* overexpression could enhance the degradation of cotton-straw lignin, which might occur due to increased extracellular laccase activity. Furthermore, some previous studies have reported the correlation between decolorization and lignin degradation of several fungal strains [7,45]. So, we estimated that *PoLac2* gene may be not only involved in the lignin degradation but also related to the decolorization of different dyes. Moreover, in the present study, we found that *PoLac5* and *PoLac12* may be involved in the formation of primordia and fruiting bodies, respectively. Therefore, further studies are needed to be conducted in the future to investigate more about the biological roles of the laccase genes in *P. ostreatus*.

## 4. Materials and Methods

### 4.1. Identification and Chromosomal Distribution of Laccase Genes in P. ostreatus

To identify all members of the *PoLac* family, three consensus domain (PF00394, PF07732, and PF07731) of the laccase hidden Markov model (HMM) was downloaded from Pfam (http://pfam.xfam.org/search/sequence). Then, by using DNATOOLS software (dnaTools, Loveland, CO, USA), these HMM profiles were used as the query in BlastP search against the publicly available genome database of *P. ostreatus* (http://genome.jgi.doe.gov/PleosPC15_2/PleosPC15_2.home.html) with an expected value (E-value) of 1 × 10^−3^. Subsequently, the Pfam database and SMART (http://smart.embl-heidelberg.de/) were used to validate that all candidate *PoLac*s contained the core domains of laccase, this step was crucial for identifying the correct number of proteins [46]. Based on the sequence alignments generated by ClustalX 1.8.1 software (http://www.clustal.org/clustal2), all potentially redundant laccase gene sequences were discarded. To understand the distribution of laccase genes in the genome, the scaffold location information of each *PoLac* gene was obtained from *P. ostreatus* genomics database and was mapped onto the scaffold using the software program Photoshop (Adobe Photoshop CS5 Extended, Adobe Systems Incorporated, CA, USA). To identify putative orthologs between two different fungi (*Pleurotus ostreatus* and *Coprinopsis cinerea*), each laccase amino acid sequences from *Pleurotus ostreatus* and *Coprinopsis cinerea* were analyzed using BLAST. Then, screening the initial result by standard (identity > 40%, e-value < 1 × 10^−10^, score > 200), two sequences were defined as orthologs if each of them was the best hit of the other [47]. The amino acid sequence of 17 laccase genes of *Coprinopsis cinerea* was obtained from NCBI GenBank (lcc1–lcc17 accession numbers: BK004111–BK004127) [2].

### 4.2. Protein Sequences and Characteristics Analysis

The Prosite ExPASy server (http://web.expasy.org/protparam/) was used to predict physicochemical characteristics of *PoLac* proteins, such as the protein molecular weight (kDa) and isoelectric point (pI). Signal peptides were predicted by SignalP 4.1 Server (http://www.cbs.dtu.dk/services/SignalP/). Transmembrane region of these laccase proteins was analyzed by TMHMM2.0 (http://www.cbs.dtu.dk/services/TMHMM-2.0/). The secondary structures of these laccase proteins were predicted by SOPMA [48] (http://npsa-pbil.ibcp.fr/cgi-bin/npsa_automat.pl?page=/NPSA/npsa_sopma.html).

### 4.3. Phylogenetic Analysis, Gene Structure, and Conserved Motifs 

Phylogenetic analysis was performed with the NJ method (p-distances substitution model, pairwise deletion and 1000 bootstrap tests) using the software package MEGA 5.1 (http://www.megasoftware.net/) based on the multiple alignments of the amino acid sequence data [10]. The online tool Gene Structure Display Server 2.0 (http://gsds.cbi.pku.edu.cn/) was used to display exon-intron layouts of each *PoLac*s DNA sequences [49]. The conserved motifs in *PoLac* proteins were analyzed using MEME (Multiple Expectation Maximization for Motif Elicitation, http://meme-suite.org/) [50] based on the following parameters: an optimum motif width of no less than 6 and no more than 200 and a maximum number of motifs of 20 [22]. Then, the conserved motifs were annotated using the Pfam database and SMART.

### 4.4. Multiple Sequence Alignment and Promoter Sequence Analysis

Based on the results of previous studies [2], the protein sequences of *Phanerochaete chrysosporium* Mco1 (GenBank No. AAO42609) and *Saccharomyces cerevisiae* ferroxidase Fet3 (GenBank No. AAA64929) were downloaded from NCBI. Multiple sequence alignment of *PoLac* proteins was carried out using DNAMAN software (Lynnon Biosoft, San Ramon, CA, USA) [31]. The 2000-bp upstream genomic DNA sequence of the start codon of each *PoLac* gene was obtained from the *P. ostreatus* genome. And then cis-elements in the promoters regions were identified using the PlantCARE database (http://bioinformatics.psb.ugent.be/webtools/plantcare/html/) SoftBerry program (http://linux1.softberry.com) and the consistent sequence information of each cis-acting elements in the article reported by Pezzella et al. [17].

### 4.5. Culture Conditions and Extraction of Enzymes

The strains *P. ostreatus* Suping No. 1 is a commercial strain provided by the Institute of Vegetable, Jiangsu Academy of Agricultural Sciences, China. The fungi were maintained at 4 °C and periodically transfer to new PDA medium.

Three disks (10 mm diameter) of the strains reactivated on dishes with solid PDA medium were transferred to the sterilized solid medium of cotton-straw. The solid culture medium contained 5 g cotton-straw powder (particle size less than 0.25 mm) and 22 mL liquid culture. The liquid culture medium contained (per liter): 20 mL of 22.0 g/L ammonium tartrate solution, 300 mL major element solutions (KH_2_PO_4_ 20 g/L, MgSO_4_·7H_2_O 13.8 g/L, CaCl_2_ 1.0 g/L, NaCl 0.6 g/L), 300 mL trace elements (MnSO_4_·H_2_O 0.35 g/L, FeSO_4_·7H_2_O 60 mg/L, CoCl_2_·6H_2_O 110 mg/L, ZnSO_4_·7H_2_O 60 mg/L, CuSO_4_·5H_2_O 95 mg/L, AlK(SO_4_)_2_·12H_2_O 6 mg/L, H_3_BO_3_ 6 mg/L, Na_2_MoO_4_·2H_2_O 6 mg/L), 60 mL of 100 mg/L VB_1_ solution and 320 mL H_2_O. Then, the culture was incubated at 25 °C in a temperature-controlled incubator for 5 days, 10 days, 15 days, 20 days, 25 days, 30 days, 40 days, 50 days, 60 days. The first 30 days is mycelium period, after 30 days of vegetative growth, change the culture conditions to make mycelium into the fruiting period. All the cultures were triplicated. A set of sterilized non-inoculated cotton-straw served as the control sample. After incubation, the mycelium attached to the solid medium of cotton-straw was removed. Then, add 15 mL of pure water to the flask, 4 °C overnight. After oscillation extraction (1 h of 200 rpm, 25 °C), the leaching solution was centrifuged at 6000 rpm for 10 min at 4 °C. The supernatant was used for measuring laccase activities. The solid residual substrates were oven-dried at 60 °C until stable weight for the Klason lignin content determination.

### 4.6. Measurement of Laccase Activity and Lignin Content 

The enzymatic activity of laccases was assayed by the oxidation of ABTS (2, 2′-azinobis-3-ethylbenzthiazoline-6-sulphonate) [51]. The reaction mixture (in a total volume of 3 mL) was slightly modified, which contained 100 μL of the enzyme solution described above, 200 μL of 0.5 mM ABTS and 2700 μL of 0.5 mM acetate buffer (pH 4.0–5.0). The reaction was performed for 3 min at room temperature before the variation in the absorbance at 420 nm was recorded. One unit of enzyme activity was defined as the amount of laccase that oxidized 1 μM ABTS per minute. Data were analyzed using SPSS with one-way ANOVA and significance was set at *p* < 0.05.

The Klason lignin content was measured by use F800 Fiber Analyzer (Hanon, Jinan, China) according to the Van Soest method [52].

### 4.7. Strains Material, Total RNA Extraction, cDNA Synthesis, and qRT-PCR

Because *P. ostreatus* is hard to grow into mature fruiting bodies on PDA medium. Mycelia of the dikaryon strain Suping No. 1 were cultivated in the sterilized solid medium of cotton-straw and PDA medium at 25 °C for 30 days, respectively. Next, opening the jars with cotton-straw medium and placing them in a chamber at 12–15 °C and 90% relative humidity, until primordium formation and mature fruiting body appeared after further days of growth. We collected samples of cotton-straw cultures on mycelial stages (10 days, 20 days, 30 days) (Figure 5a), primordia, fruiting body at the young and maturation stages (Figure 5b–d). PDA medium cultures on 10 days, 20 days, and 30 days. All samples were immediately frozen in liquid nitrogen.

Total RNA from each sample was extracted by using an RNAprep pure plant kit (Tiangen, Beijing, China). Then, the DNase-treated RNA was reverse transcribed into cDNA using an RT reagent kit with gDNA Eraser (TaKaRa, Shanghai, China). The PCR primers (Shown in supplementary Table S1) were designed for qRT-PCR by using Primer Premier 5.0 software (Premier Biosoft, Palo Alto, CA, USA). qRT-PCR experiments were performed using a CFX96 (Bio-Rad, Hercules, CA, USA) instrument to examine the gene expression in different cDNA samples. The reaction mixtures (20 μL volumes) contained the following: 10 μL of SYBR Premix Ex *Taq*II (2x), 2 μL of template cDNA, 0.8 μL of forward and reverse primers, and water. Each reaction was performed in triplicate. PCR amplification conditions were as follows: 50 °C for 2 min and 95 °C for 30 s, followed by 40 cycles of 95 °C for 15 s, 60 °C for 20 s, and 72 °C for 20 s. The relative expression levels of target genes were calculated using the 2^−ΔΔCT^ method [53], with the *sar* gene used as an internal control [54].

### 4.8. Construction of Lac2 Overexpressing Plasmid Vector

The *PoLac2* sequence was found in the JGI genome database of PC15 v2.0 (http://genome.jgi.doe.gov/PleosPC15_2/PleosPC15_2.home.html) as sequence ID 1067328. High-fidelity PCR to obtain the full-length *PoLac2* cDNA using L2-F(GAAGATCTGATGGTGCTCTCTACTAAGCTCGCTGCTC) and L2-R(GGACTAGTCTGGAACTCGGGAGCGAGGCCATCATAAG) as primers (underlined nucleotides in the primers are the restriction sites for BglII and SpeI respectively), and the cDNA from the mycelial stage as a template. The PCR product was then purified and ligated into the pMD18-T vector (TAKARA, Beijing, China) for sequencing by biological companies (Sangon Biotech, Shanghai, China). Finally, the recombinant pMD18-T plasmid, which contained the specific sequence of *PoLac2*, was digested with BglII and SpeI and this digested fragment was inserted into the same sites of pCambia 1304-SDI-GPD-HygR vector which reconstruction on the basis of pCambia1304 vectors (GenBank: AF234300.1) by our laboratory (reconstruction plasmid map shown in Additional file: Figure S2). We named the validated recombinant plasmid as pCambia 1304-SDI-GPD-HygR-*PoLac2* and the pCambia 1304-SDI-GPD-HygR-*PoLac2* vector contained the endogenous promoter of the *sdi* gene.

### 4.9. Fungal Transformation

*P. ostreatus* strain Suping No. 1 was cultured in potato-dextrose broth (PDB) medium at 25 °C and 150 rpm for 3–4 days and used for transformation. *P. ostreatus* mycelium pellet was transformed using the ATMT method [55,56] as previously described with slight modifications. The transformation procedure was compared with the method described by [55]. The recombinant expression plasmid (pCambia 1304-SDI-GPD-HygR-*PoLac2*) were transformed into the competent strain by electroporation with the voltage, capacitance and resistance was 2400 V, 25 μF, and 200 Ω, respectively. Next, 250 μL *A. tumefaciens* strain EHA105 harboring pCambia 1304-SDI-GPD-HygR-*PoLac2* vector was grown in 25 mL of LB medium containing 50 μg/mL rifampin and 50 μg/mL kanamycin for 24–48 h at 28 °C on a rotatory shaker (200 rpm) until the broth color turned to orange. Bacterial cells were harvested by centrifugation at 5000 rpm for 10 min, the supernatant was discarded and the cells were resuspended in fresh induction medium (IM, PDB medium + 200 μmol/L AS) to an OD_600_ of 0.4–0.5, and then grown at 28 °C on 100 rpm for 3–6 h. *P. ostreatus* mycelium pellets were immersed into IM of pre-induced *A. tumefaciens* at 25 °C for 20–30 min. After that, the superfluous bacteria fluid on mycelia pellets were removed with sterilized filter paper. Then, mycelia pellets were placed on co-cultivation medium (Co-IM, PDA medium + 200 μmol/L AS) plate at 25 °C. After co-cultivation for 3 days, the newly grown hyphae were picked and transferred to PDA plates with 200 μg/mL cefotaxime and 50 μg/mL hygromycin B to selecting potential *P. ostreatus* transformants. Wild-type fungus was used as the control for selection and screening was performed 3 times.

### 4.10. Analysis of the Transformants

The genomic DNA was extracted from the mycelia of the transformants and wild-type strain and the presence of *gusA* was confirmed by PCR using the following forward primer: 5′-GTCCTGTAGAAACCCCAACCCGTGA-3′ and reverse primer: 5′-TTTGCCTCCCTGCTGCGGTTTTTCA-3′. Transgenic and wild-type samples were stained by using histochemical assays kit (Real-Times, Beijing, China) at 37 °C overnight. After staining, the samples were bleached with several washes of 70% ethanol and then photographed. Qualitative assay of the wild-type and transformants laccase activity was performed by guaiacol agar plates (PDA medium supplemented with 0.04% individually sterilized guaiacol). The *PoLac2* expression levels in the mycelium period were detected using qRT-PCR and the primers used are same as those listed in Table S2. The laccase activity and the Klason lignin content of the wild-type and transformants grown in the cotton-straw medium were measured by the previously described method.

## Figures and Tables

**Table 1:** The predicted and tallied physiochemical properties of 12 putative laccases genes in *P. ostreatus*.

Gene Name	Sequences ID	Number of Amino Acids (aa)	Molecular Weight (kDa)	pI	Signal Peptide Cleavage Site	Intron Number
*PoLac1*	1043420	532	57.57525	5.14	23–24	19
*PoLac2*	1067328	522	57.47591	5.66	19–20	21
*PoLac3*	1102751	541	58.86968	6.27	21–22	17
*PoLac4*	1077328	522	57.35740	5.00	21–22	10
*PoLac5*	1094975	630	69.36803	4.96	19–20	9
*PoLac6*	1113032	533	57.92248	6.27	20–21	15
*PoLac7*	1077468	507	55.62609	6.08	19–20	11
*PoLac8*	1106925	534	58.69680	7.78	25–26	15
*PoLac9*	1089733	529	56.58007	4.53	23–24	19
*PoLac10*	1089723	533	56.79672	4.68	23–24	19
*PoLac11*	1043488	543	59.41680	5.23	18–19	16
*PoLac12*	1094965	513	55.28401	5.03	23–24	16

## Supplementary Materials

The following are available online. Tables S1–S4; Figures S1 and S2.
