# Supplementary material for: Systematic Analysis of the Pleurotus ostreatus Laccase Gene (PoLac) Family and Functional Characterization of PoLac2 Involved in the Degradation of Cotton-Straw Lignin
Source: Molecules. 2018 Apr 11;23(4):880. doi: 10.3390/molecules23040880 (PMC6017272; doi:10.3390/molecules23040880)
Supplement: Supplementary file 1 [file molecules-23-00880-s001.pdf]

Article

Systematic Analysis of the *Pleurotus ostreatus* Laccase Gene (*PoLac*) Family and Functional Characterization of *PoLac2* Involved in the Degradation of Cotton-Straw Lignin

Xiaoyu Jiao [1,†], Guoqing Li [1,2,†], Yan Wang [1], Fan Nie [2], Xi Cheng [1], Muhammad Abdullah [1], Yi Lin [1] and Yongping Cai [1,*]

[1] School of Life Sciences, Anhui Agricultural University, No. 130, Changjiang West Road, Hefei 230036, China; jxy2015@ahau.edu.cn (X.J.); liguoqing1976@163.com (G.L.); wangyanahau@163.com (Y.W.); cxzp1114@163.com (X.C.); abdullahpadana@hotmail.com (M.A.); linyi1957@126.com (Y.L.)

[2] Horticultural Institute, Anhui Academy of Agricultural Sciences, Hefei 230031, China; fan.n@163.com

* Correspondence: ypcaiah@163.com; Tel.:+86-551-6578-6137

† These authors contributed equally to this work.

Table S1. The predicted secondary structures of 12 putative laccase identified in *P. ostreatus.*

Gene name	α-Helix (%)	Extended strand (%)	β-Turn (%)	Random coils (%)
PoLac1	13.16	34.77	11.47	40.60
PoLac2	16.28	29.69	11.88	42.15
PoLac3	13.68	33.83	12.57	39.93
PoLac4	12.07	30.27	11.11	46.55
PoLac5	22.38	27.14	10.16	40.32
PoLac6	11.44	32.46	10.88	45.22
PoLac7	16.57	28.99	12.23	42.21
PoLac8	16.67	30.90	11.61	40.82
PoLac9	14.37	32.14	11.34	42.16
PoLac10	11.82	32.27	11.63	44.28
PoLac11	15.29	29.10	9.02	46.59
PoLac12	13.26	33.33	12.09	41.33

Table S2. *PoLac* gene-specific primers used for qRT-PCR analysis.

Gene	Transcript identification no. (PC15)	Primer name	Primer sequence (5′- 3′)
Lac1	1043420	Fw	CGGTACATCTTAGCACCCAATG
		Rv	GGACAGGGCTCGCTGGTT
Lac2	1067328	Fw	CATGACCTTGCTCCCCAC
		Rv	GATGACAGCGTAAGGGAC
Lac3	1102751	Fw	GTAAGAAGTGCAGGAAGCTCAACA
		Rv	CCCGTTCCGGTGGAAAC
Lac4	1077328	Fw	CCGTGACGTGCCATACGATG
		Rv	GGTCGAAGGGTGCGTGCGG
Lac5	1094975	Fw	CGCATTTGCCGCTTTCTT
		Rv	GGTGACTAGGACTGAGTATCTC
Lac6	1113032	Fw	GTACAACTACGAAAACCCCG
		Rv	CAAGGTCAAGATGCCAGT
Lac7	1077468	Fw	GTTGATAGCCTCCAGATCTTCG
		Rv	GTAGGATGGCGGAGTTGATG
Lac8	1106925	Fw	CATTGGCTGTGACTCGAA
		Rv	GGATCAGAGAATAGCGTTGG
Lac9	1089733	Fw	GCTTACCGGTGTCCTCGTG
		Rv	GTCATATAGGAAGCTGTTC
Lac10	1089723	Fw	GTTCCTTTCCTTCCACCAAC
		Rv	CTCCAGCTCGTAAATGCTAC
Lac11	1043488	Fw	GTACTTAGGGCCGTACTTG
		Rv	GTCGGTATTCATGGTCGTG
Lac12	1094965	Fw	CTATACCGCCCACCTTGCCTCG
		Rv	GGAAATTGACCGCTAGACGAC
sar1	1052294	Fw	GGATAGTCTTCCTCGTCGATAG
		Rv	GGGTGCGTCAATCTTGTTAC

Table S3. Detailed information of the 20 motifs in 12 putative *P. ostreatus* laccase proteins.

Motif	Width	Best Possible Match	Domain
1	101	PTPDSTLINGLGRYAGGPTSPLAVINVERGKRYRIRLISISCDPNYTFSIDGHSMTI IEADGENTQPLEVDSIQIFAGQRYSFVLNANQAIGNYWIRANP	Cu-oxidase
2	143	EDPTTTESNSTNPLLETBLVPLENPGAPGPPVPGGADVNINLAFAFDFTTFEFTI NGVPFVPPTAPVLLQILSGASTAASLLPAGSVYTLPPNKVVEJSM	*
3	59	KGDRFQJNVINZLSDTTMLKTTSIHWHGLFQKGTNWADGPAFVTQCPIVPGDS FLYDFK	Cu-oxidase_3
4	57	PDQAGTFWYHSHLSTQYCDGLRGPFVVYDPNDPHKSLYDVDDESTVJTLEDW YHVPA	Cu-oxidase_3
5	22	CHIDWHLEJGLAVVFAEDPAST	Cu-oxidase_2
6	15	DNVTIRFVTDNPGPW	*
7	21	LGSPGFEGGINSAILRYKGAP	*
8	21	GPTADLHIVNKVIAPDGFSRS	*
9	15	NPVPAAWDDLCPIYB	*
10	11	GTYPGPLIQGN	*
11	20	MFPGARILATLTLALHLLHG	*
12	16	AGKHPFHLHGHKFDIV	Cu-oxidase_2
13	10	ALSDEEKGGI	*
14	29	KYYRFRLINISCRPFFFFWIDGHDFDVIE	Cu-oxidase
15	11	KLLMGTNAJGR	*
16	7	HAQGQFF	*
17	6	NPPRRD	*
18	8	IYFAQRAS	*
19	6	ANKLIE	*
20	8	ELNPPIKE	*

Table S4. Orthologous analyses of laccase genes between *P. ostreatus* and *C. cinerea*.

Numble	orthologous genes	
1	PoLac1	Cclcc11
2	PoLac2	Cclcc17
3	PoLac3	Cclcc10
4	PoLac4	Cclcc7
5	PoLac6	Cclcc7
6	PoLac7	Cclcc10
7	PoLac8	Cclcc6
8	PoLac9	Cclcc7
9	PoLac10	Cclcc11
10	PoLac11	Cclcc9
11	PoLac12	Cclcc11

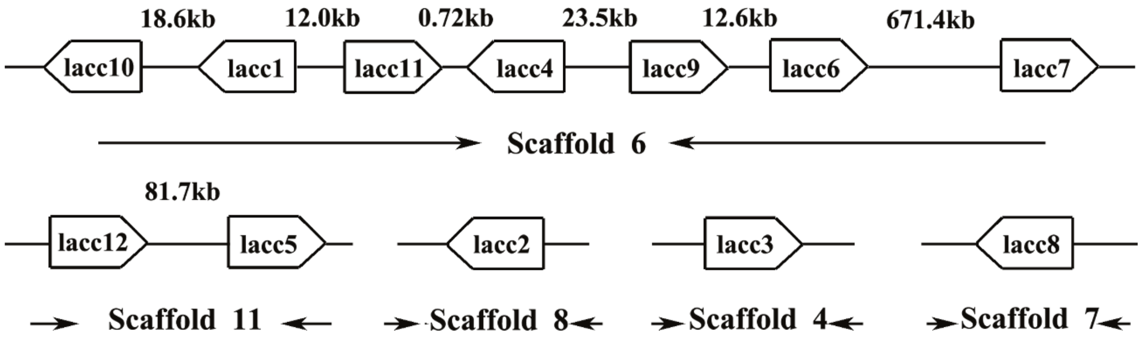

Figure S1. Distribution of laccase genes in the genome of *P. ostreatus* (PC 15). Arrow-shaped boxes indicate the laccase genes, while the direction of arrow shows the direction for each gene. The numerical values above the line show the distances (bp) between the laccase genes.

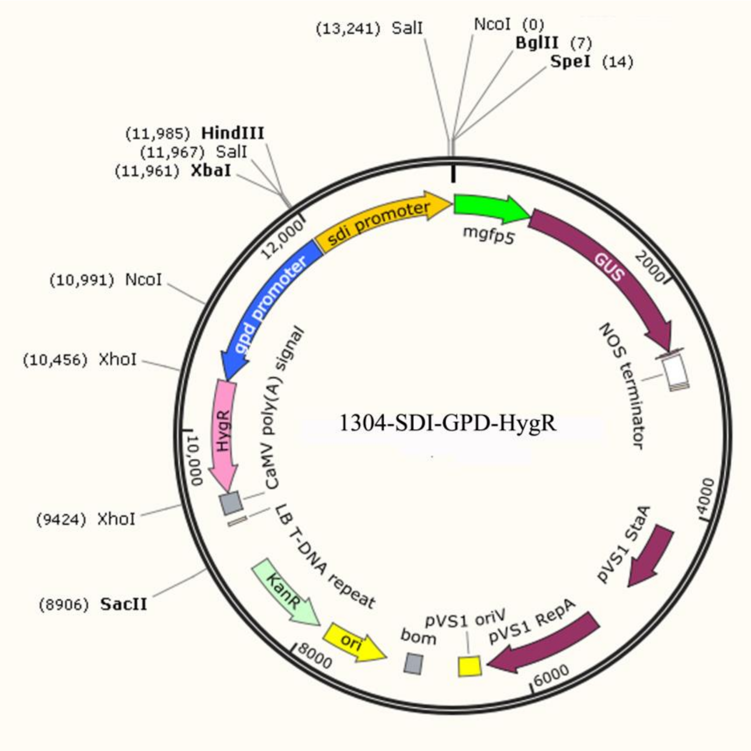

Figure S2. Structures of modified binary vector pCambia 1304-SDI-GPD-HygR. *gusA* and target gene are under the influence of sdi promoter, while hygromycin resistance (*HygR*) gene is expressed using GPD promoter.
